# Weak Phd2-Hif-1α Affinity Coupled with High Basal Expression Is Predicted to Enhance HIF Pathway Flexibility in Nile Tilapia (*Oreochromis niloticus*)

**DOI:** 10.3390/ani16101561

**Published:** 2026-05-21

**Authors:** Junli Yan, Xianzong Wang, Dan Liu, Jing Song, Shaozhen Liu, Qing Liu, Zhongbao Guo

**Affiliations:** 1 College of Urban and Rural Construction, Shanxi Agricultural University, Taigu District, Jinzhong 030801, China; y_jl2020@163.com; 2 College of Animal Science, Shanxi Agricultural University, Taigu District, Jinzhong 030801, China; xianzong_wang@126.com (X.W.); ivan06_jasper@163.com (D.L.); songjingoak@163.com (J.S.); shmily8316@126.com (S.L.); 3 Shanxi Provincial Key Laboratory of Livestock and Poultry Genetic Resources Exploration and Biotechnology Breeding, Shanxi Agricultural University, Taigu District, Jinzhong 030801, China; 4 Guangxi Academy of Fishery Sciences, 8 Qingshan Road, Nanning 530021, China

**Keywords:** hypoxia-inducible factor, prolyl hydroxylase domain protein, loop region, expression, dissolved oxygen, temperature, mitochondrion, oxygen-labile α subunit

## Abstract

To explore the molecular basis of hypoxia tolerance variation within euteleosts, we compared the hypoxia-inducible factor (HIF) pathways of the highly tolerant Nile tilapia (*Oreochromis niloticus*) and the hypoxia-sensitive rainbow trout (*Oncorhynchus mykiss*). Evolutionary analysis revealed that Nile tilapia possesses single copies of Hif-1α and prolyl hydroxylase domain protein 2 (Phd2), whereas rainbow trout retains two and three copies, respectively. The Leu-X-X-Leu-Ala-Pro (LXXLAP) motifs in the oxygen-dependent degradation (ODD) domain of Hif-1α and the interacting loop region of Phd2 are highly conserved, indicating a conserved core mechanism for regulating Hif-1α stability. However, differences in charged residue composition flanking the Phd2 loop (e.g., fewer positively charged residues in Nile tilapia) were identified. Molecular dynamics simulations revealed that the complex formed by Nile tilapia Phd2 and the Hif-1α LXXLAP motif was unstable across physiological temperatures, suggesting potential impairment of the catalytic geometry compatible with hydroxylation and elevated normoxic Hif-1α stability. In contrast, the corresponding complexes in rainbow trout were more stable, particularly at low temperatures. Expression profiling revealed that Nile tilapia tissues, including the heart, maintain higher basal expression of glycolytic genes, may help support energy production during hypoxia. Our findings indicate that a weakened protein interaction and high constitutive expression is predicted to enhance HIF pathway responsiveness, potentially priming vital tissues for glycolytic energy production and may contribute to this species’ hypoxia tolerance.

## Simple Summary

Nile tilapia is a fish known for its ability to survive in low-oxygen water, yet it lacks the extra copies of oxygen-sensing genes that other hardy fish, like carp, use to achieve this tolerance. This study explored how tilapia achieves this using only a single set of these genes. Computational models and gene expression analyses suggest a different strategy: the interaction between the key oxygen-sensing proteins is predicted to be weaker in tilapia than in sensitive species like rainbow trout, while the baseline production of these proteins is higher. We hypothesize that this “tuned” sensitivity is predicted to provide greater flexibility in the oxygen response pathway. As a result, vital organs such as the heart may be primed to rapidly increase energy production through glycolysis when oxygen levels drop, helping the fish cope with hypoxic stress.

## 1. Introduction

Dissolved oxygen (DO) levels in aquatic environments frequently fall below the physiological requirements of fish, making hypoxia a pervasive challenge in both natural waters and aquaculture systems [1,2]. Fishes have evolved diverse strategies to cope with hypoxic stress, including behavioral responses (e.g., aquatic surface respiration, avoidance), cardiovascular adjustments (e.g., increased stroke volume), and hematological adaptations (e.g., modulation of hemoglobin-oxygen affinity) [3,4,5]. Hypoxia tolerance is species-, life stage-, and context-dependent, and its enhancement may involve physiological trade-offs such as reduced growth performance or altered energy metabolism [6]. For hypoxia-tolerant species such as common carp (*Cyprinus carpio*) [7], aeration alone is often sufficient even under high-density farming. In contrast, for hypoxia-sensitive species such as rainbow trout (*Oncorhynchus mykiss*) [8], supplemental liquid oxygen is typically required to meet their high oxygen demand. Consequently, enhancing the hypoxia tolerance of high-value species may be economically attractive, although such improvements often involve physiological trade-offs and remain challenging to achieve in practice.

The hypoxia-inducible factor (HIF) pathway is the central mechanism for cellular oxygen sensing and response in animals [9,10]. The functional HIF complex is a heterodimer composed of an oxygen-regulated α subunit (HIF-1α, HIF-2α, or HIF-3α) and a constitutively expressed β subunit (also known as aryl hydrocarbon receptor nuclear translocator, ARNT) [11]. Under normoxic conditions, prolyl hydroxylase domain proteins (PHD1–3) hydroxylate specific proline residues within the oxygen-dependent degradation (ODD) domain of HIF-αs [12]. This modification enables recognition by the von Hippel–Lindau (VHL) tumor suppressor protein, leading to ubiquitination and subsequent proteasomal degradation of HIF-αs [9]. Under hypoxia, PHD activity is suppressed, allowing HIF-α subunits to accumulate [13]. The stabilized HIF-α subunit then dimerizes with ARNT. It subsequently recruits transcriptional coactivators (e.g., p300/CBP) via the C-terminal transactivation domain (C-TAD) to form an active transcription complex [13,14]. This complex binds to hypoxia-response elements (HREs) in the promoters of target genes, upregulating the expression of hundreds of genes involved in processes such as glycolysis, angiogenesis, and erythropoiesis, thereby improving cellular adaptation to low oxygen [15,16,17]. In addition to this canonical oxygen-dependent regulation, HIF-1α activity can also be modulated by oxygen-independent pathways, including growth factor signaling and the mTOR/AMPK axis, which influence its translation [9,13]. Nevertheless, the oxygen-dependent hydroxylation of HIF-1α by PHDs remains the primary and most rapid mechanism governing its protein stability.

Although the core components of the HIF pathway (HIF-1α, ARNT, PHD2, and VHL) are highly conserved from nematodes to mammals, the complexity of the pathway has gradually increased during evolution [10,18]. Whole-genome duplication (WGD) events have been a major driver of this increased complexity. The paralogs of HIF-1α and PHD2, for example, originated from the two rounds of WGD that occurred in the common ancestor of vertebrates [19,20]. In fishes, additional WGD events—including the teleost-specific genome duplication (TGD) and subsequent lineage-specific duplications—have led to further expansion of paralogs for core HIF pathway components in certain fish lineages [21,22,23,24]. In a recent comparative study, we found that otomorphs (e.g., cyprinids and catfish) generally retain both Hif-1α paralogs (Hif-1αa and Hif-1αb) originating from the TGD, whereas euteleosts lost Hif-1αb early in their evolution [25]. Furthermore, key variations are universally found in the Leu-X-X-Leu-Ala-Pro (LXXLAP) motifs of the Hif-1αa proteins in otomorphs. These variations are predicted to impair binding by Phd2, which may increase the stability of the Hif-1αa proteins under normoxia. This subfunctionalization of Hif-1α paralogs is hypothesized to contribute to vital otomorphs tissues, including the heart, to rapidly upregulate glycolysis, contributing to their generally high hypoxia tolerance.

While the duplication-subfunctionalization strategy appears to have contributed to hypoxia tolerance in otomorphs, it is not the only evolutionary route to this phenotype. Nile tilapia (*Oreochromis niloticus*), a member of the euteleosts, also exhibits remarkable hypoxia tolerance [26]. Notably, the tilapia lineage has not undergone additional WGD events since the euteleost common ancestor, and it appears to retain only single copies of Hif-1α and Phd2. This contrasts with the paralog retention observed in otomorphs and raises the possibility: tilapia may have achieved its hypoxia tolerance not through “hardware redundancy” (gene duplication and subfunctionalization), but through regulatory and biochemical fine-tuning of a single-copy HIF pathway. If so, what specific molecular adjustments might underlie such optimization?

For a single-copy system to increase its functional efficiency, at least two non-mutually exclusive routes are available: (1) modulation of the intrinsic biochemical properties of the proteins themselves—for instance, through amino acid substitutions that alter protein–protein interaction affinities; and (2) quantitative adjustment of gene expression levels, which can be achieved via evolutionary changes in promoter or enhancer elements. If Nile tilapia has evolved elevated hypoxia tolerance without gene duplication, it is plausible that one or both of these routes may have been utilized. Based on this reasoning, we propose the following hypothesis: the hypoxia tolerance of Nile tilapia arises from a “tuned” single-copy HIF system characterized by weakened Phd2-Hif-1α interaction affinity and elevated constitutive expression of core components. To test this hypothesis, we conducted a comparative analysis between Nile tilapia and the hypoxia-sensitive rainbow trout, integrating evolutionary genomics, molecular dynamics simulations, and tissue expression profiling.

## 2. Materials and Methods

The analytical workflow used here builds upon the computational framework established in our previous study [25]. For clarity and reproducibility, the specific parameters, software versions, and analytical approach employed in the present comparative study of Nile tilapia and rainbow trout are detailed in the subsequent Section 2.1, Section 2.2, Section 2.3, Section 2.4 and Section 2.5.

### 2.1. Identification of Homologs

We used the human HIF-1α protein sequence (GenBank: NP_001230013.1) as a query to identify its homologs in teleost fishes. Non-redundant protein sequences for mouse (*Mus musculus*), chicken (*Gallus gallus*), and seven fish species were retrieved from the NCBI RefSeq FTP site (https://ftp.ncbi.nlm.nih.gov/genomes/refseq/, accessed on 23 November 2025). The corresponding relationship between protein accessions and gene IDs was obtained from the “gene2accession” file (downloaded from https://ftp.ncbi.nlm.nih.gov/gene/DATA/, accessed on 23 November 2025). For genes with multiple isoforms, we selected the longest protein variant. A local BLASTP search (using ncbi-blast-2.15.0+) was performed against these sequences with the human HIF-1α query, using a maximum of 1000 target sequences and an e-value cutoff of 1 × 10^−5^. Hits with pairwise query coverage > 30% were retained. This threshold was selected after evaluating hit recovery across a range of coverage cutoffs (Supplementary Table S1), balancing comprehensive capture of divergent teleost homologs against the inclusion of spurious hits. The query and retained sequences were combined and aligned using MAFFT (v7.427) with the L-INS-i algorithm [27]. A maximum-likelihood phylogenetic tree was then inferred from this alignment using RAxML (v8.2.8) under the GAMMA model of rate heterogeneity, with an automatically selected substitution model and 500 bootstrap replicates [28].

To distinguish orthologs from paralogs, we identified chromosomal neighbors for all candidate genes by sorting gene start positions within chromosomes using data from the “gene2accession” file. Candidate sequences that were not orthologous to the human query were identified and removed. Orthology was strictly assessed based on two criteria: (1) phylogenetic tree topology, where true orthologs were expected to form a monophyletic clade distinct from other HIF-α family members, and (2) conserved synteny, evaluated by examining the genomic neighbors of each candidate gene. An initial, unfiltered phylogenetic tree illustrating the identification and removal of non-orthologous sequences is provided as Supplementary Figure S1. The remaining orthologous sequences were realigned, and a final phylogenetic tree was generated from this refined alignment.

Using this same pipeline, we also identified homologs of PHD2 (GenBank: NP_071334.1) and known HIF-1α downstream target genes in mouse, chicken, and seven fish species. Orthology assignments were cross-validated using OrthoFinder v2.5.5 (-S diamond_ultra_sens) [29] on the complete proteomes of all ten species. The results were consistent with our manual analysis for PHD2. For HIF-1α, the automated pipeline separated the divergent cyprinid Hif-1αa paralogs into a distinct orthogroup (Supplementary Figure S2), confirming the superior sensitivity of our phylogeny-informed synteny-guided approach.

### 2.2. Identification of Conserved Domains

We performed a batch conserved domain search (CD-search; https://www.ncbi.nlm.nih.gov/Structure/bwrpsb/bwrpsb.cgi, accessed on 10 December 2025) using default parameters with an e-value threshold of 0.01 to identify domains in all selected protein sequences. When overlapping domain predictions occurred for a given sequence, we discarded the hit with the higher (i.e., less significant) e-value. Following this filtering, all retained domain annotations possessed e-values substantially lower than 1 × 10^−5^, indicating high-confidence predictions. To visualize domains in relation to the phylogenetic tree, we mapped the start and end positions of identified domains onto the corresponding coordinates in the multiple sequence alignment. Based on previous literature [30,31] and manual inspection of the alignments, we annotated the positions of the LXXLAP motifs in HIF-1α proteins and specific loop regions in PHD2 proteins. CDD integrates models from Pfam, SMART, COG, and other sources; thus, CD-search results represent a consensus across multiple annotation databases [32]. Key domain annotation differences were additionally cross-checked against InterPro (https://www.ebi.ac.uk/interpro/, accessed on 20 April 2026) [33].

### 2.3. Protein Complex Modeling

Structures of Phd2.Hif-1α_CODD_ complexes (Phd2 in complex with the Hif-1α C-terminal ODD domain, see Supplementary Table S2 for details) were predicted using AlphaFold (v2.3.1) [34,35]. As a structural template, we used a crystal structure of PHD2 bound to a HIF-1α peptide, 2-oxoglutarate (2OG), and a Mn(II) ion (PDB ID: 5L9B). Each AlphaFold-predicted complex was aligned to this template via its Phd2 component. Structural alignment confirmed that the predicted catalytic cores, including the 2OG/Mn(II) binding pocket, were highly congruent with the template (RMSD < 1 Å for active site residues; see Supplementary Figure S3 for representative alignment). Subsequently, the Hif-1α peptide in the predicted model was trimmed using PyMOL (v2.5.0) [36]. This process allowed us to transfer the coordinates of the 2OG molecule and the metal ion from the template to the corresponding positions in the predicted complexes. Predicted complexes, however, should be interpreted cautiously, as AlphaFold is not optimized for peptide docking.

We added ACE and NME caps to the N- and C-termini of each peptide, respectively. To model the physiologically active state, we replaced the crystallographic Mn(II) ion with Fe(II). Hydrogen atoms were added to the 2OG ligand at pH 7.0 using Avogadro (v1.2.0) [37]. Force field parameters for 2OG were generated using CGenFF (v2.5) [38]; all associated penalty scores were below 10, indicating the derived topology and parameters were reliable.

### 2.4. Molecular Dynamics Simulations

We conducted molecular dynamics (MD) simulations using GROMACS (2023.1) [39,40]. Simulations employed the CHARMM36 all-atom force field and the TIP3P water model [41]. Each complex was solvated in a periodic rhombic dodecahedral box, ensuring a minimum distance of 12 Å between the solute and the box boundary. We added ions to neutralize the system charge and to achieve a physiological concentration of 0.15 M NaCl. All systems were energy-minimized using the steepest descents algorithm until the maximum force fell below 1000.0 kJ mol^−1^ nm^−1^. System equilibration involved two phases: (1) a 1000 ps NVT ensemble simulation (constant Number of particles, Volume, and Temperature) to stabilize temperature at 303 K (30 °C) or 289 K (16 °C), followed by (2) a 1000 ps NPT ensemble simulation (constant Number of particles, Pressure, and Temperature) to stabilize pressure at 1 bar. Production MD simulations were run for 200 ns per system, with coordinates saved every 10 ps. All MD simulations employed a 2 fs timestep. Bond lengths involving hydrogen atoms were constrained using the LINCS algorithm. The temperature was maintained using a velocity-rescaling thermostat (τ_T = 0.1 ps), and pressure was controlled using the Parrinello-Rahman barostat (τ_P = 2.0 ps) with isotropic coupling. Long-range electrostatic interactions were treated using the Particle Mesh Ewald (PME) method, with a real-space cutoff of 1.2 nm. Van der Waals interactions were calculated with a force-switch modifier from 1.0 to 1.2 nm. Periodic boundary conditions were applied in all three dimensions (xyz). For each condition, we performed six independent simulation replicas, starting from the energy minimization step. We calculated interatomic distances using standard GROMACS utilities. Binding free energy was calculated for representative complexes using gmx_MMPBSA (v1.6.4) [42].

### 2.5. Gene Expression Analyses

We analyzed tissue-specific gene expression patterns in Nile tilapia and rainbow trout. We identified relevant BioProjects for each species, prioritizing those that sequenced multiple tissues from adult fish (see Supplementary Table S3). Using the BioProject accessions, we queried the SRA Run Selector (https://www.ncbi.nlm.nih.gov/Traces/study/, accessed on 3 December 2025) to obtain download links for the raw RNA-seq data. Compressed read files were converted to FASTQ format using fasterq-dump (SRA Toolkit v3.0.5). We then used fastp (v0.23.4) with default parameters to perform quality control, removing low-quality reads, adapters, and contaminants [43]. Reference transcriptomes and genomes for both species were downloaded from the NCBI RefSeq FTP site (https://ftp.ncbi.nlm.nih.gov/genomes/refseq/, accessed on 3 December 2025). We built genome indices in SAF format for each species using salmon index [44]. Transcript abundance for each sample was quantified with salmon quant (v1.10.0). The relationship between transcript and gene identifiers was established using the NCBI “gene2accession” file. Finally, we summed the transcripts per million (TPM) values for all splice variants of a gene to obtain a single gene-level TPM value. To account for potential batch effects arising from different BioProjects, we fitted a linear mixed model for each gene’s log-transformed TPM values using statsmodels (v0.12.2) [45], with tissue type as a fixed effect and BioProject ID as a random effect. For each gene, we constructed the appropriate linear contrast from the estimated fixed-effect coefficients to obtain its marginal mean expression in each tissue. The associated standard errors were derived from the coefficient covariance matrix. These adjusted tissue means, which represent the expected expression after accounting for batch effects, were used for downstream correlation analysis. Correlations were calculated using Pearson’s method, and *p*-values were adjusted for multiple testing using the Benjamini–Hochberg False Discovery Rate (FDR) procedure. Despite batch correction, cross-study comparisons remain subject to residual technical variation arising from differences in library preparation and sequencing platforms.

## 3. Results

### 3.1. Nile Tilapia Possesses Fewer Copies of Hif-1α and Phd2 than Rainbow Trout

**Figure 1:**
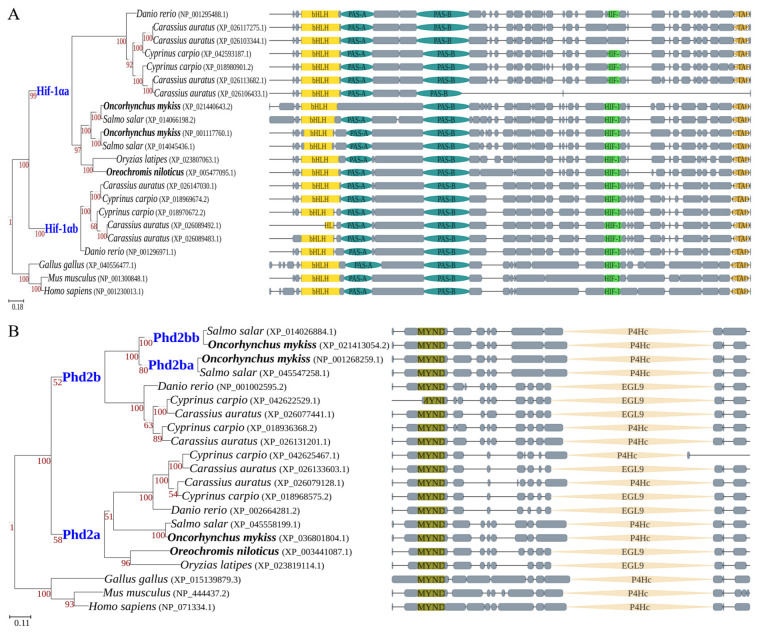
Phylogenetic analysis and domain architecture of HIF-1α (**A**) and PHD2 (**B**) in different lineages. The maximum likelihood trees (**left**) depict the evolutionary relationships of HIF-1α (or PHD2) sequences from seven fish species, chicken, mouse, and human. Species names of Nile tilapia and rainbow trout are highlighted in bold. Hif-1αa/αb and Phd2a/b paralogs (shown in blue) originated from the teleost-specific genome duplication (TGD). Their presence or absence in subsequent lineages indicates gene retention or loss, respectively. Schematics (**right**) show the domain organization of corresponding sequences. Colorful blocks represent conserved domains predicted by CD-search; gray blocks represent aligned regions; horizontal lines indicate gaps in the sequence alignment. Domain abbreviations are defined as follows: bHLH, basic helix-loop-helix; PAS (-A and -B), Per-Arnt-Sim; HIF-1, hypoxia-inducible factor-1; HIF-1a_CTAD, HIF-1α C terminal transactivation domain; zf-MYND, MYND finger; P4Hc, prolyl 4-hydroxylase alpha subunit homologues; EGL9, egl-9 family hypoxia-inducible factor prolyl hydroxylases.

To identify orthologs, we performed BLASTP searches using human HIF-1α and PHD2 protein sequences as queries against seven fish species and reconstructed phylogenetic trees. Orthology was confirmed based on tree topology and conserved synteny. The teleost-specific genome duplication (TGD) generated two copies each of Hif-1α and Phd2, as retained in zebrafish (*Danio rerio*) (Figure 1). However, selective pressure often leads to the loss of redundant copies. Among euteleosts, Nile tilapia and medaka retain only Hif-1αa and Phd2a (the TGD-derived paralogs), having lost Hif-1αb and Phd2b. In contrast, rainbow trout and Atlantic salmon, while also having lost Hif-1αb, retained Phd2b. Furthermore, in these salmonids, both Hif-1αa and Phd2b are present as two copies, consistent with the salmonid-specific genome duplication [23].

### 3.2. Sequence Divergence in Key Functional Domains

**Figure 2:**
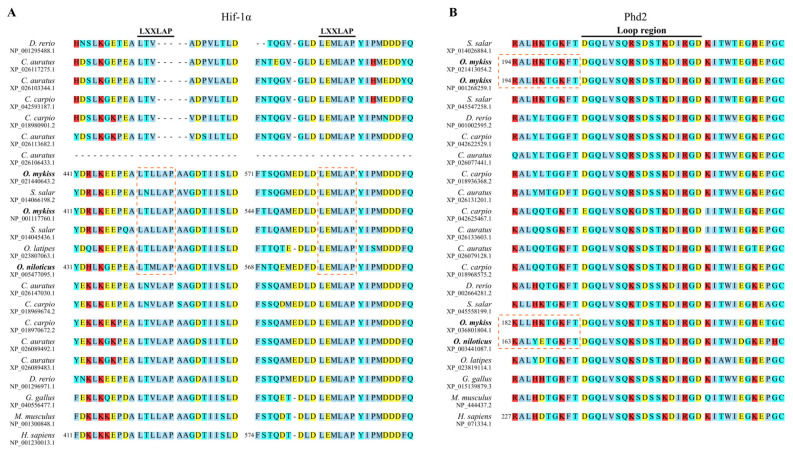
Multiple sequence alignment of key regions in HIF-1α and PHD2. (**A**) Alignment of HIF-1α sequences surrounding the two LXXLAP motifs; (**B**) Alignment of PHD2 sequences surrounding the loop region. Species names of Nile tilapia and rainbow trout are highlighted in bold. Background colors denote amino acid properties: nonpolar (sky blue), polar neutral (cyan), basic (red), acidic (yellow). For sequences of rainbow trout, Nile tilapia, and human, numbers on the left side indicate the starting position within the full-length protein. Species names of Nile tilapia and rainbow trout are highlighted in bold. Conserved LXXLAP motifs and key differences in charged residues flanking the Phd2 loop are highlighted with rectangular dashed boxes.

Beyond copy number variation, we identified sequence divergence in key functional domains of Hif-1α and Phd2 between rainbow trout and Nile tilapia. In rainbow trout, one of the two Hif-1αa paralogs lacks a canonical PAS-A domain (Figure 1A). The two LXXLAP motifs, critical for regulating Hif-1α stability, were highly conserved in all three Hif-1α proteins from both species (Figure 2A). This contrasts with cyprinids (e.g., zebrafish, common carp, goldfish (*Carassius auratus*)), where key variations in these motifs are common and previously reported to affect stability [25].

The PHD2 protein contains two conserved domains, the second of which confers hydroxylase activity. Interestingly, the domain annotation differed among lineages: in humans, mice, chickens, and salmonids, it is identified as a P4Hc domain (cdd: smart00702), whereas in Nile tilapia, medaka, and zebrafish, it is annotated as an EGL9 domain (cdd: COG3751). These domains belong to distinct superfamilies according to NCBI’s Conserved Domain Database [32]. However, this distinction was not detected by InterPro, suggesting that the two annotations may reflect database-specific classification rather than fundamental structural divergence. Within this catalytic domain, a key loop region directly interacts with the Hif-1α LXXLAP motifs. While the loop region itself is conserved, its flanking sequences exhibit marked differences (Figure 2B). Specifically, all three rainbow trout Phd2 proteins possess four positively charged residues adjacent to the left side of the loop, whereas the Nile tilapia Phd2 has only two (vs. four) positively charged residues flanking a negatively charged residue. This disparity in local electrostatic potential may influence interaction properties (see Discussion).

### 3.3. Weaker Predicted Binding Affinity of Nile Tilapia Phd2 for Hif-1A

**Figure 3:**
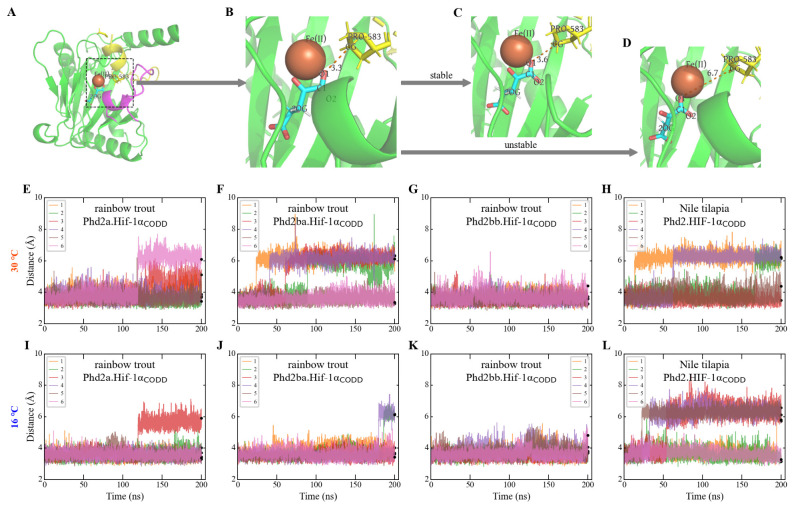
Molecular dynamics simulation analysis of Phd2-Hif-1α complex stability. (**A**–**D**) Representative structural snapshots illustrating stable vs. unstable binding in simulations of the Nile tilapia Phd2a.Hif-1α_CODD_. Phd2 is colored green, the CODD peptide yellow, and the key interacting loop region within Phd2 is magenta. (**A**,**B**) are taken from the first frame, while (**C**,**D**) are from the final frame of the 200 ns simulation. The Pro_CODD_-CG to 2OG-O1 distances in (**B**), (**C**) and (**D**) are 3.3 Å, 3.6 Å, and 6.7 Å, respectively. (**E**–**L**) Time-dependent changes in the Pro_CODD_-CG to 2OG-O1 distance for six independent replicas of each complex. This distance serves as a metric for binding stability. A sustained increase in this distance beyond ~3.5 Å, often leading to complete separation (>6 Å), was considered indicative of complex instability. (**E**–**H**) were simulated at 30°C, while (**I**–**L**) were simulated at 16 °C.

Given the sequence divergence near the interaction interface, we hypothesized a differential binding affinity between Phd2 and Hif-1α in the two species, which would impact hydroxylation efficiency. To test this, we performed molecular dynamics (MD) simulations. Analysis of the human PHD2-HIF-1α complex (PDB: 5L9B) and predicted protein complexes revealed that the distance between the target proline CG atom (Pro_CODD_) and the O1 atom of the co-substrate 2-oxoglutarate (2OG) is critical for hydroxylation (Figure 3A,B). This is consistent with structural evidence that the target proline C–H bond must project toward the metal center for hydroxylation to occur and that only the correct prolyl ring conformation enables a productive complex [31]. In our simulations, the initial CG–O1 distance in all complexes was ~3.3 Å (Figure 3B), within the upper limit of a hydrogen bonding distance (2.5–3.5 Å). Given that hydrogen bonding networks between substrate and cosubstrate are essential for maintaining the correct pre-catalytic geometry in 2OG-dependent dioxygenases [46], the CG–O1 distance probes not only catalytic geometry but also local interaction strength at the active site. Therefore, we used the CG–O1 distance as a metric for binding stability in our simulations. We modeled four complexes: three from rainbow trout (Phd2a.Hif-1α_CODD_, Phd2ba.Hif-1α_CODD_, and Phd2bb.Hif-1α_CODD_) and one from Nile tilapia (Phd2.Hif-1α_CODD_). Six independent simulation replicas were run for each complex at two temperatures: 30 °C (near-optimal for Nile tilapia) and 16 °C (near-optimal for rainbow trout). Cα RMSD analysis confirmed that the overall conformation of Phd2 remained stable throughout the simulations (Supplementary Figure S4), with the magnitude of fluctuations comparable to those reported for similar systems [47]. Total energy trajectories further confirmed that all systems reached thermodynamic equilibrium well before the analysis window (Supplementary Figure S5).

At 30 °C, the Nile tilapia Phd2 complex exhibited high instability, with Pro_CODD_–2OG dissociation occurring in four of six replicas within 200 ns (earliest at <20 ns) (Figure 3H). Among rainbow trout complexes, the Phd2ba complex showed instability similar to the Nile tilapia complex (Figure 3F), whereas the Phd2bb complex remained stable across all replicas (Figure 3G).

The PHD2-HIF-1α interaction is known to be weak (high *K_m_*) [13]. We therefore examined the complexes at lower temperature (16 °C) to assess potential stabilization. At 16 °C, the rainbow trout Phd2a and Phd2ba complexes showed increased stability, evidenced by the decreased frequency and delayed time of occurrence of dissociations (Figure 3I,J). In stark contrast, the Nile tilapia complex did not exhibit increased stability, with dissociation still occurring in four replicas (Figure 3L). Also at 16 °C, the difference in dissociation frequency between the grouped rainbow trout complexes (3 events in 18 replicas) and the Nile tilapia complex (4 events in 6 replicas) was statistically significant (Fisher’s exact test, *p* = 0.038).

We also calculated the binding free energy between Phd2 and the Hif-1α short peptide for representative complexes and performed root mean square fluctuation (RMSF) analysis at 30 °C. The binding free energy did not show a clear correlation with the CG–O1 distance (Supplementary Figure S6). This is not unexpected: the binding free energy encompasses contributions from all inter-residue contacts across the entire PHD2–CODD interface, many of which are remote from the catalytic center, whereas the CG–O1 distance specifically probes the local geometry and interaction at the active site. In a weak-affinity, highly dynamic system such as the PHD2–HIF-1α complex, catalytic competence may be determined primarily by the integrity of the active-site geometry rather than by the overall binding strength [47]. Furthermore, RMSF analysis indicated high flexibility in the Phd2 loop region across all complexes but did not reveal distinct differences between species (Supplementary Figure S7). While these additional metrics did not differentiate the complexes, their results align with the weak and transient interaction characteristics of these complexes. The CG–O1 distance, by directly probing the local interaction geometry at the catalytic center, serves as an informative indicator for assessing the integrity of the active site across different complexes in this context.

### 3.4. Divergent Expression Profiles of HIF Pathway Genes

**Figure 4:**
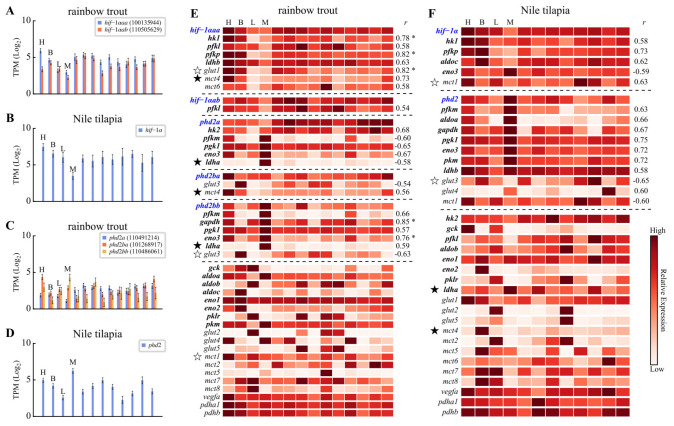
Tissue expression profiles and co-expression analysis of HIF pathway genes in rainbow trout and Nile tilapia. Tissue codes: H, heart; B, brain; L, liver; M, muscle. (**A**–**D**) Expression levels (log-transformed TPM) of *hif-1α* and *phd2* paralogs across tissues. Error bars represent the standard error of the marginal means (Prediction_SE) derived from the linear mixed model. (**E**,**F**) Heatmaps displaying relative expression profiles of core HIF pathway genes. The Pearson correlation coefficient (*r*) between the expression profile of a downstream gene and a *hif-1α* or *phd2* gene (in blue) is shown if the correlation is nominally significant (*p* < 0.05); correlations that remain significant after FDR correction (FDR < 0.05) are additionally marked with an asterisk (“*”). Genes encoding key glycolytic enzymes are highlighted in bold. The positions of the *ldha* and *mct4* genes in the heatmaps are prominently marked with pentagram symbols (“★”), whereas the *mct1*, *glut1*, and *glut3* genes are marked with empty pentagram symbols (“☆”). TPM, transcripts per million.

The MD simulations suggested that Nile tilapia Phd2 has a weaker capacity to regulate Hif-1α stability than rainbow trout Phd2 paralogs. This is consistent with the possibility of greater normoxic accumulation of Hif-1α and potentially stronger induction of downstream genes in Nile tilapia (see Supplementary Table S4 for target gene details). RNA-Seq data from 18 BioProjects (see Supplementary Table S3) supported this inference. In vital tissues (heart, brain, liver, muscle), the expression level of single-copy *hif-1α* and *phd2* in Nile tilapia was generally higher than the summed expression of their multiple paralogs in rainbow trout (Figure 4A–D; note that expression values in the figure are log-transformed). Correlation analysis revealed that in both species, the expression profiles of a few glycolytic genes correlated significantly with *hif-1α*. In contrast, more glycolytic genes showed significant co-expression with Nile tilapia *phd2* and rainbow trout *phd2bb* (Figure 4E,F). Rainbow trout *phd2a* expression correlated negatively with some glycolytic genes. These co-expression patterns are consistent with a potential association between specific Phd2 paralogs and the glycolytic program, in line with the known role of Phd2 in regulating Hif-1α. Based on their co-expression profiles with downstream glycolytic genes (Figure 4E,F), rainbow trout *phd2bb* was more similar to the single-copy Nile tilapia *phd2* than to its trout paralogs. This functional similarity inferred from expression data contrasts with the MD simulation results at 30 °C, where the Phd2bb complex was markedly more stable than the Nile tilapia Phd2 complex (Figure 3G,H). Collectively, these observations suggest that the different Phd2 paralogs in rainbow trout have undergone complex functional divergence.

Further analysis of key metabolic genes revealed marked differences. Lactate dehydrogenase A (LDHA), which directs pyruvate to lactate, is typically muscle-specific. Rainbow trout *ldha* followed this pattern, whereas Nile tilapia *ldha* was highly expressed in all tissues examined (Figure 4E,F). For lactate efflux under high intracellular lactate concentration, monocarboxylate transporter 4 (MCT4) is typically utilized. In rainbow trout, *mct4* was highly expressed in the heart and brain but low elsewhere. In Nile tilapia, *mct4* expression was largely restricted to the brain. Instead, Nile tilapia showed high, ubiquitous expression of *mct1*, whose profile correlated significantly with *hif-1α*. Differences were also evident in glucose transporters (GLUTs): Nile tilapia constitutively expressed both *glut1* and *glut3* across tissues, while rainbow trout showed constitutive expression only of *glut1*.

## 4. Discussion

In the preceding Results section, we presented findings from our comparative genomic, molecular dynamics, and transcriptomic analyses, along with direct interpretations of these data. Here, we synthesize these observations to propose a cohesive model for how the HIF pathway is tuned in Nile tilapia. Under hypoxic stress, cells need to reduce oxygen consumption while maintaining ATP production. A shift from oxidative phosphorylation to glycolysis addresses both needs, but the end product must be lactate to regenerate NAD^+^ [5,48,49,50,51]. This step is catalyzed by lactate dehydrogenase (LDH), a tetramer of LDHA and/or LDHB subunits [52,53]. LDHA-rich complexes favor lactate production, while LDHB-rich complexes favor oxidation. In strictly aerobic tissues like the mammalian heart, LDHB dominates [51,54,55]. The fish heart, however, has substantial energy demands and must be capable of metabolic flexibility during severe hypoxia [5,56,57]. We therefore paid particular attention to *ldha* expression patterns in our comparative analysis, alongside other core components of the glycolytic pathway.

Our related work in otomorphs (e.g., zebrafish, goldfish) revealed constitutive, high *ldha* expression, particularly in the heart [25]. This aligns with the high normoxic stability and heart-enriched expression of their Hif-1αa proteins, a key transcriptional activator of *ldha*. In otomorphs, the two TGD-derived Hif-1α paralogs have subfunctionalized: Hif-1αa, though often lowly expressed, is stable and poised to rapidly induce glycolytic genes; Hif-1αb is highly expressed but oxygen-labile, accumulating during sustained hypoxia to amplify the adaptive response. This duplication-divergence mechanism has been proposed to contribute to the strong hypoxia tolerance of many otomorphs. However, the direct causal link between these molecular features and organismal hypoxia tolerance has not been experimentally validated.

**Figure 5:**
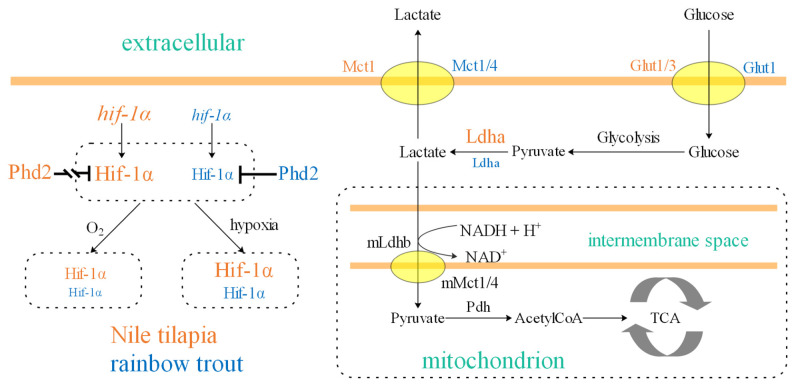
Proposed model for hypoxia tolerance in Nile tilapia mediated by a tuned HIF pathway. Key features compared to rainbow trout: (1) Weaker Phd2-Hif-1α interaction affinity; (2) Higher basal expression of *phd2* and *hif-1α*. This combination is predicted to result in elevated cellular Hif-1α concentration under normoxia, which can further increase during hypoxia. This may pre-adapt tissues (e.g., heart) by maintaining high levels of glycolytic enzymes like Ldha, could facilitate a more rapid glycolytic response upon hypoxic exposure. Additionally, Nile tilapia may rely more on Mct1 for lactate export. Relative gene expression (mRNA) and protein concentrations (inferred from this study) are schematically represented by font and symbol sizes. Core metabolic pathways are adapted from [50].

Rainbow trout, despite possessing multiple Hif-1α and Phd2 copies (Figure 1), has not evolved a comparable subfunctionalization-based mechanism. The two Hif-1α paralogs in rainbow trout retain highly similar LXXLAP motifs in their CODD and NODD domains (Figure 2A), suggesting they remain functionally redundant in terms of oxygen-dependent degradation, rather than undergoing the clear subfunctionalization seen in otomorphs. Nile tilapia, in contrast, appears to employ a distinct strategy for hypoxic preparedness, centered on tuning protein–protein interaction affinity and expression levels. First, the weak Phd2-Hif-1α interaction affinity (Figure 3) coupled with high constitutive *hif-1α* expression (Figure 4B,D) is consistent with a model in which normoxic Hif-1α levels are elevated, priming tissues like the heart for glycolysis (evidenced by high basal *ldha*). Second, the concomitantly high *phd2* expression may provide the capacity for a dynamic regulatory range. Under normoxia, substantial hydroxylation and degradation still occur, preventing excessive HIF activity. During hypoxia, the already weak interaction is further suppressed. This may facilitate more rapid Hif-1α accumulation and potent transcriptional activation (Figure 5).

The need to export lactate also appears to be addressed differently. While MCT4 (high *K_m_* for lactate) typically handles lactate efflux under glycolytic conditions [50,58], Nile tilapia exhibited minimal *mct4* expression outside the brain. Instead, *mct1* was highly and ubiquitously expressed and correlated with *hif-1α*. Notably, expression of Nile tilapia *mct1* has been reported to be upregulated by dietary sodium lactate [59]. We hypothesize that in Nile tilapia, Mct1 may play a compensatory role, fulfilling the lactate export function typically served by MCT4. This hypothesis remains to be tested, for instance through functional characterization of the Nile tilapia Mct1 protein.

The strategy employed by Nile tilapia relies on fine-tuning a single-copy HIF system, which contrasts with the duplication-driven subfunctionalization of otomorphs. Despite this difference, the outcome is analogous: both are predicted to enhance the effective concentration of Hif-1α under normoxia, leading to a basal priming of downstream glycolytic genes. This priming enables a rapid response to energy crisis induced by hypoxia. Moreover, this priming appears to be not systemic but preferentially associated with a few critical tissues (e.g., the heart), thereby achieving an optimal balance between effectiveness and energetic economy. Our study demonstrates that, in core regulatory pathways, fine-tuning the interactions between existing components (“qualitative optimization”) is an equally important source of evolutionary plasticity as increasing gene copy number (“quantitative expansion”). Whether similar regulatory tuning could be achieved in hypoxia-sensitive euteleosts remains an open question for future investigation.

It is important to note that the “enhancement” of HIF pathway flexibility proposed in our title and model is a key prediction derived from our integrated computational analyses—including the predicted weak Phd2-Hif-1α interaction, high constitutive expression, and consequent elevated normoxic priming of glycolytic genes. This study has several limitations inherent to its computational nature. First, the key conclusion of weakened Phd2-Hif-1α interaction affinity and its physiological consequence is inferred from molecular dynamics simulations and correlative expression analyses, not from direct experimental measurement. Direct experimental validation through in vitro binding assays or in vivo functional studies is lacking and represents a necessary next step. Second, the transcriptomic data were mined from public repositories. Although we applied batch effect correction using a linear mixed model to account for BioProject-level variation, and we selected control samples from adult fish, some unmeasured heterogeneity may persist. Third, the MD simulations, while informative, were conducted on simplified complexes and cannot fully replicate the intracellular environment.

## 5. Conclusions

In conclusion, our integrated analysis is consistent with a model in which Nile tilapia’s hypoxia tolerance is associated with a “tuned” single-copy HIF pathway, characterized by computationally inferred weak Phd2-Hif-1α interaction affinity and elevated constitutive gene expression. Our model posits that this strategy is predicted to enhance pathway flexibility (a conceptual synthesis rather than a measured parameter) and may contribute to poising key tissues for glycolytic demand. This represents a regulatory fine-tuning strategy that contrasts with the duplication-driven subfunctionalization observed in otomorphs. While our findings provide a testable model for the plasticity of the conserved HIF pathway, the predicted molecular interactions and their physiological consequences warrant direct experimental validation. This work highlights how fine-tuning existing molecular components can be an equally important source of evolutionary innovation as gene duplication and offer a framework for future experimental investigation of HIF pathway tuning in hypoxia-tolerant fishes.

## Data Availability

The original contributions presented in the study are included in the article/its Supplementary Materials; further inquiries can be directed to the corresponding authors.

## Supplementary Materials

The following supporting information can be downloaded at: https://www.mdpi.com/article/10.3390/ani16101561/s1, Table S1: Number of BLASTP hits retained at different pairwise query coverage thresholds for HIF1A and PHD2. Table S2: Protein components of complexes used for MD simulations. Table S3: BioProjects of Nile tilapia and rainbow trout used for gene expression analyses. Table S4: Detailed information of downstream genes in four fish species. Figure S1. An initial unfiltered phylogenetic tree showing the identification and removal of non-orthologous sequences. Figure S2. Cross-validation of orthology assignments for HIF-1α and PHD2 using OrthoFinder. Figure S3. Structural alignment of AlphaFold-predicted Phd2.Hif-1α_CODD_ complexes with the crystallographic template. Figure S4. Cα RMSD of Phd2 proteins during MD simulations. Figure S5. Total energy stabilization during MD simulations. Figure S6. Relationship between calculated binding free energy and CG–O1 distance over time. Figure S7. Root mean square fluctuation (RMSF) of Cα atoms in CODD peptides and Phd2.
