# Supplementary material for: Weak Phd2-Hif-1α Affinity Coupled with High Basal Expression Is Predicted to Enhance HIF Pathway Flexibility in Nile Tilapia (Oreochromis niloticus)
Source: Animals (Basel). 2026 May 21;16(10):1561. doi: 10.3390/ani16101561 (PMC13203693; doi:10.3390/ani16101561)
Supplement: Supplementary file 1 [file animals-16-01561-s001.zip › tilapia supplementary material v0.3.pdf]

Supplementary Material

Weak Phd2-Hif-1α Affinity Coupled with High Basal Expression Enhances HIF Pathway Flexibility in Nile Tilapia (*Oreochromis niloticus*)

Junli Yan[1,†], Xianzong Wang[2,3,†], Dan Liu[2], Jing Song[2,3], Shaozhen Liu[2,3], Qing Liu[2,3,*], and Zhongbao Guo[4,*]

[1] College of Urban and Rural Construction, Shanxi Agricultural University, Taigu District, Jinzhong 030801, China

[2] College of Animal Science, Shanxi Agricultural University, Taigu District, Jinzhong 030801, China

[3] Shanxi Provincial Key Laboratory of Livestock and Poultry Genetic Resources Exploration and Biotechnology breeding, Shanxi Agricultural University, Taigu District, Jinzhong 030801, China

[4] Guangxi Academy of Fishery Science, 8 Qingshan Road, Nanning 530021, China

*** Correspondence**: liuqing_sxau@126.com; guozhongbaono1@163.com

[†] These authors contributed equally to this work.

Table S1. Number of BLASTP hits retained at different pairwise query coverage thresholds for HIF1A and PHD2.

Protein	TaxID	90%	80%	70%	60%	50%	40%	35%	30%	25%	20%
HIF1A	8022	0	0	0	1	2	6	6	**7**	18	29
	8030	0	0	0	2	2	7	7	**7**	19	29
	8090	0	0	0	1	2	4	4	**4**	8	12
	8128	0	0	0	1	1	4	4	**4**	7	14
	7955	0	0	1	1	1	5	5	**7**	11	17
	7957	0	0	3	4	10	13	13	**19**	30	44
	7962	0	0	2	3	5	10	10	**13**	19	33
	10090	0	1	1	1	1	2	3	**3**	8	12
	9031	0	1	1	1	2	2	2	**2**	4	10
PHD2	8022	0	0	3	3	5	6	7	**7**	7	7
	8030	0	0	1	3	5	6	7	**7**	7	7
	8090	0	0	1	1	2	2	3	**3**	3	3
	8128	0	0	1	1	2	3	3	**3**	3	3
	7955	0	0	0	0	3	4	4	**4**	4	4
	7957	0	0	2	3	6	9	9	**9**	9	9
	7962	0	0	2	2	3	7	8	**8**	8	9
	10090	0	1	1	1	2	3	3	**3**	3	3
	9031	0	0	1	1	2	2	2	**2**	2	2

Table S2. Protein components of complexes used for MD simulations.

Organism	Protein (accession No.)	Region for structure prediction (aa)	Region for MD simulation (aa)	Complex name[a]
Rainbow trout	Hif-1αa (NP_001117760.1)	539-579	552-569<br>LD**LEMLAP**YIPMDDDFQL	Phd2a.Hif-1α$_{CODD}$
	Phd2a (XP_036801804.1)	116-378	142-366	
Rainbow trout	Hif-1αa (NP_001117760.1)	539-579	552-569<br>LD**LEMLAP**YIPMDDDFQL	Phd2ba.Hif-1α$_{CODD}$
	Phd2ba (NP_001268259.1)	125-390	156-378	
Rainbow trout	Hif-1αa (NP_001117760.1)	539-579	552-569<br>LD**LEMLAP**YIPMDDDFQL	Phd2bb.Hif-1α$_{CODD}$
	Phd2bb (XP_021413054.2)	125-390	156-378	
Nile tilapia	Hif-1α (XP_005477095.1)	563-603	576-593<br>FD**LEMLAP**YIPMDDDFQ	Phd2.Hif-1α$_{CODD}$
	Phd2 (XP_003441087.1)	103-359	125-347	

[a] Although we did not include the names of 2OG and Fe(II), they were present in each final complex for MD simulations.

Table S3. BioProjects of Nile tilapia and rainbow trout used for gene expression analyses.

Organism	Bioproject No.	Average bases per run/G	Sampled tissues
rainbow trout	PRJEB37848	4.5	brain, eye, gill, gut, head_kidney, heart, kidney, liver, muscle, pyloric_caeca, skin, spleen
rainbow trout	PRJEB57191	9.7	brain, fast_muscle_tissue, gill_filament, gonad_male, head_kidney, liver, terminal_part_of_digestive_tract
rainbow trout	PRJNA1226994	7.1	brain, heart, kidney, liver, muscle, ovary, spleen, testis
Nile tilapia	PRJDB6721	6.7	brain, heart, spleen
Nile tilapia	PRJNA1017631	8	ovary, testis
Nile tilapia	PRJNA1074672	6.9	ovary, testis
Nile tilapia	PRJNA1120617	8.1	heart
Nile tilapia	PRJNA1129205	7	intestine, liver
Nile tilapia	PRJNA1199930	12.7	muscle
Nile tilapia	PRJNA1235842	8.7	skin
Nile tilapia	PRJNA532787	20.8	head_kidney, spleen
Nile tilapia	PRJNA612550	5.9	liver
Nile tilapia	PRJNA720254	7.3	brain, muscle
Nile tilapia	PRJNA846796	7.5	stomach
Nile tilapia	PRJNA910236	7.9	intestine
Nile tilapia	PRJNA953605	8.4	gill
Nile tilapia	PRJNA976970	8.8	gill
Nile tilapia	PRJNA996550	6.4	head_kidney

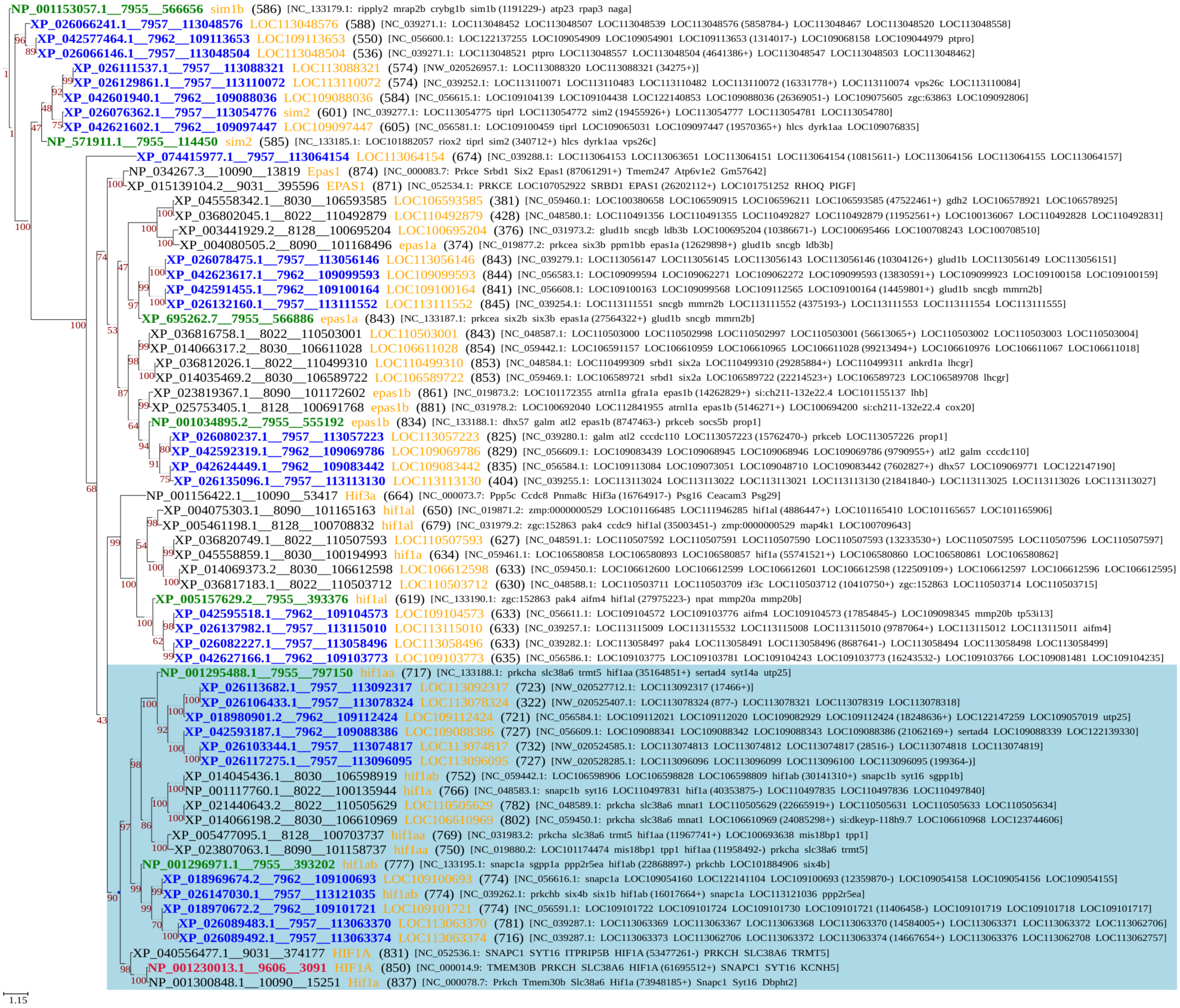

NP_001153057.1__7955__566656 sim1b (586) [NC_133179.1: ripply2 mrap2b crybg1b sim1b (1191229-) atp23 rpap3 naga]
XP_026066241.1__7957__113048576 LOC113048576 (588) [NC_039271.1: LOC113048452 LOC113048507 LOC113048539 LOC113048576 (5858784-) LOC113048467 LOC113048520 LOC113048558]
XP_042577464.1__7962__109113653 LOC109113653 (550) [NC_056600.1: LOC122137255 LOC109054909 LOC109054901 LOC109113653 (1314017-) LOC109068158 LOC109044979 ptpro]
XP_026066146.1__7957__113048504 LOC113048504 (536) [NC_039271.1: LOC113048521 ptpro LOC113048557 LOC113048504 (4641386+) LOC113048547 LOC113048503 LOC113048462]
XP_026111537.1__7957__113088321 LOC113088321 (574) [NW_020526957.1: LOC113088320 LOC113088321 (34275+)]
XP_026129861.1__7957__113110072 LOC113110072 (574) [NC_039252.1: LOC113110071 LOC113110483 LOC113110482 LOC113110072 (16331778+) LOC113110074 vps26c LOC113110084]
XP_042601940.1__7962__109088036 LOC109088036 (584) [NC_056615.1: LOC109104139 LOC109104438 LOC122140853 LOC109088036 (26369051-) LOC109075605 zgc:63863 LOC109092806]
XP_026076362.1__7957__113054776 sim2 (601) [NC_039277.1: LOC113054775 tiprl LOC113054772 sim2 (19455926+) LOC113054777 LOC113054781 LOC113054780]
XP_042621602.1__7962__109097447 LOC109097447 (605) [NC_056581.1: LOC109100459 tiprl LOC109065031 LOC109097447 (19570365+) hlcs dyrk1aa LOC109076835]
NP_571911.1__7955__114450 sim2 (585) [NC_133185.1: LOC101882057 riox2 tiprl sim2 (340712+) hlcs dyrk1aa vps26c]
XP_074415977.1__7957__113064154 LOC113064154 (674) [NC_039288.1: LOC113064153 LOC113063651 LOC113064151 LOC113064154 (10815611-) LOC113064156 LOC113064155 LOC113064157]
NP_034267.3__10090__13819 Epas1 (874) [NC_000083.7: Prkce Srbd1 Six2 Epas1 (87061291+) Tmem247 Atp6v1e2 Gm57642]
XP_015139104.2__9031__395596 EPAS1 (871) [NC_052534.1: PRKCE LOC107052922 SRBD1 EPAS1 (26202112+) LOC101751252 RHOQ PIGF]
XP_045558342.1__8030__106593585 LOC106593585 (381) [NC_059460.1: LOC100380658 LOC106590915 LOC106596211 LOC106593585 (47522461+) gdh2 LOC106578921 LOC106578925]
XP_036802045.1__8022__110492879 LOC110492879 (428) [NC_048580.1: LOC110491356 LOC110491355 LOC110492827 LOC110492879 (11952561+) LOC100136067 LOC110492828 LOC110492831]
XP_003441929.2__8128__100695204 LOC100695204 (376) [NC_031973.2: glud1b sncgb ldb3b LOC100695204 (10386671-) LOC100695466 LOC100708243 LOC100708510]
XP_004080505.2__8090__101168496 epas1a (374) [NC_019877.2: prkcea six3b ppm1bb epas1a (12629898+) glud1b sncgb ldb3b]
XP_026078475.1__7957__113056146 LOC113056146 (843) [NC_039279.1: LOC113056147 LOC113056145 LOC113056143 LOC113056146 (10304126+) glud1b LOC113056149 LOC113056151]
XP_042623617.1__7962__109099593 LOC109099593 (844) [NC_056583.1: LOC109099594 LOC109062271 LOC109062272 LOC109099593 (13830591+) LOC109099923 LOC109100158 LOC109100159]
XP_042591455.1__7962__109100164 LOC109100164 (841) [NC_056608.1: LOC109100163 LOC109099568 LOC109112565 LOC109100164 (14459801+) glud1b sncgb mmrn2b]
XP_026132160.1__7957__113111552 LOC113111552 (845) [NC_039254.1: LOC113111551 sncgb mmrn2b LOC113111552 (4375193-) LOC113111553 LOC113111554 LOC113111555]
XP_695262.7__7955__566886 epas1a (843) [NC_133187.1: prkcea six2b six3b epas1a (27564322+) glud1b sncgb mmrn2b]
XP_036816758.1__8022__110503001 LOC110503001 (843) [NC_048587.1: LOC110503000 LOC110502998 LOC110502997 LOC110503001 (56613065+) LOC110503002 LOC110503003 LOC110503004]
XP_014066317.2__8030__106611028 LOC106611028 (854) [NC_059442.1: LOC106591157 LOC106610959 LOC106610965 LOC106611028 (99213494+) LOC106610976 LOC106611067 LOC106611018]
XP_036812026.1__8022__110499310 LOC110499310 (853) [NC_048584.1: LOC110499309 srbd1 six2a LOC110499310 (29285884+) LOC110499311 ankrd1a lhcgr]
XP_014035469.2__8030__106589722 LOC106589722 (853) [NC_059469.1: LOC106589721 srbd1 six2a LOC106589722 (22214523+) LOC106589723 LOC106589708 lhcgr]
XP_023819367.1__8090__101172602 epas1b (861) [NC_019873.2: LOC101172355 atrnl1a gfra1a epas1b (14262829+) si:ch211-132e22.4 LOC101155137 lhb]
XP_025753405.1__8128__100691768 epas1b (881) [NC_031978.2: LOC100692040 LOC112841955 atrnl1a epas1b (5146271+) LOC100694200 si:ch211-132e22.4 cox20]
NP_001034895.2__7955__555192 epas1b (834) [NC_133188.1: dhx57 galm atl2 epas1b (8747463-) prkceb socs5b prop1]
XP_026080237.1__7957__113057223 LOC113057223 (825) [NC_039280.1: galm atl2 cccdc110 LOC113057223 (15762470-) prkceb LOC113057226 prop1]
XP_042592319.1__7962__109069786 LOC109069786 (829) [NC_056609.1: LOC109083439 LOC109068945 LOC109068946 LOC109069786 (9790955+) atl2 galm cccdc110]
XP_042624449.1__7962__109083442 LOC109083442 (835) [NC_056584.1: LOC109113084 LOC109073051 LOC109048710 LOC109083442 (7602827+) dhx57 LOC109069771 LOC122147190]
XP_026135096.1__7957__113113130 LOC113113130 (404) [NC_039255.1: LOC113113024 LOC113113022 LOC113113021 LOC113113130 (21841840-) LOC113113025 LOC113113026 LOC113113027]
NP_001156422.1__10090__53417 Hif3a (664) [NC_000073.7: Ppp5c Ccdc8 Pnma8c Hif3a (16764917-) Psg16 Ceacam3 Psg29]
XP_004075303.1__8090__101165163 hif1al (650) [NC_019871.2: zmp:0000000529 LOC101166485 LOC111946285 hif1al (4886447+) LOC101165410 LOC101165657 LOC101165906]
XP_005461198.1__8128__100708832 hif1al (679) [NC_031979.2: zgc:152863 pak4 ccdc9 hif1al (35003451-) zmp:0000000529 map4k1 LOC100709643]
XP_036820749.1__8022__110507593 LOC110507593 (627) [NC_048591.1: LOC110507592 LOC110507591 LOC110507590 LOC110507593 (13233530+) LOC110507595 LOC110507596 LOC110507597]
XP_045558859.1__8030__100194993 hif1a (634) [NC_059461.1: LOC106580858 LOC106580893 LOC106580857 hif1a (55741521+) LOC106580860 LOC106580861 LOC106580862]
XP_014069373.2__8030__106612598 LOC106612598 (633) [NC_059450.1: LOC106612600 LOC106612599 LOC106612601 LOC106612598 (122509109+) LOC106612597 LOC106612596 LOC106612595]
XP_036817183.1__8022__110503712 LOC110503712 (630) [NC_048588.1: LOC110503711 LOC110503709 if3c LOC110503712 (10410750+) zgc:152863 LOC110503714 LOC110503715]
XP_005157629.2__7955__393376 hif1al (619) [NC_133190.1: zgc:152863 pak4 aifm4 hif1al (27975223-) npat mmp20a mmp20b]
XP_042595518.1__7962__109104573 LOC109104573 (633) [NC_056611.1: LOC109104572 LOC109103776 aifm4 LOC109104573 (17854845-) LOC109098345 mmp20b tp53i13]
XP_026137982.1__7957__113115010 LOC113115010 (633) [NC_039257.1: LOC113115009 LOC113115532 LOC113115008 LOC113115010 (9787064+) LOC113115012 LOC113115011 aifm4]
XP_026082227.1__7957__113058496 LOC113058496 (633) [NC_039282.1: LOC113058497 pak4 LOC113058491 LOC113058496 (8687641-) LOC113058494 LOC113058498 LOC113058499]
XP_042627166.1__7962__109103773 LOC109103773 (635) [NC_056586.1: LOC109103775 LOC109103781 LOC109104243 LOC109103773 (16243532-) LOC109103766 LOC109081481 LOC109104235]
NP_001295488.1__7955__797150 hif1aa (717) [NC_133188.1: prkcha slc38a6 trmt5 hif1aa (35164851+) sertad4 syt14a utp25]
XP_026113682.1__7957__113092317 LOC113092317 (723) [NW_020527712.1: LOC113092317 (17466+)]
XP_026106433.1__7957__113078324 LOC113078324 (322) [NW_020525407.1: LOC113078324 (877-) LOC113078321 LOC113078319 LOC113078318]
XP_018980901.2__7962__109112424 LOC109112424 (721) [NC_056584.1: LOC109112021 LOC109112020 LOC109082929 LOC109112424 (18248636+) LOC122147259 LOC109057019 utp25]
XP_042593187.1__7962__109088386 LOC109088386 (727) [NC_056609.1: LOC109088341 LOC109088342 LOC109088343 LOC109088386 (21062169+) sertad4 LOC109088339 LOC122139330]
XP_026103344.1__7957__113074817 LOC113074817 (732) [NW_020524585.1: LOC113074813 LOC113074812 LOC113074817 (28516-) LOC113074818 LOC113074819]
XP_026117275.1__7957__113096095 LOC113096095 (727) [NW_020528285.1: LOC113096096 LOC113096099 LOC113096100 LOC113096095 (199364-)]
XP_014045436.1__8030__106598919 hif1ab (752) [NC_059442.1: LOC106598906 LOC106598828 LOC106598809 hif1ab (30141310+) snapc1b syt16 sgpp1b]
NP_001117760.1__8022__100135944 hif1a (766) [NC_048583.1: snapc1b syt16 LOC110497831 hif1a (40353875-) LOC110497835 LOC110497836 LOC110497840]
XP_021440643.2__8022__110505629 LOC110505629 (782) [NC_048589.1: prkcha slc38a6 mnat1 LOC110505629 (22665919+) LOC110505631 LOC110505633 LOC110505634]
XP_014066198.2__8030__106610969 LOC106610969 (802) [NC_059450.1: prkcha slc38a6 mnat1 LOC106610969 (24085298+) si:dkeyp-118h9.7 LOC106610968 LOC123744606]
XP_005477095.1__8128__100703737 hif1aa (769) [NC_031983.2: prkcha slc38a6 trmt5 hif1aa (11967741+) LOC100693638 mis18bp1 tpp1]
XP_023807063.1__8090__101158737 hif1aa (750) [NC_019880.2: LOC101174474 mis18bp1 tpp1 hif1aa (11958492-) prkcha slc38a6 trmt5]
NP_001296971.1__7955__393202 hif1ab (777) [NC_133195.1: snapc1a sgpp1a ppp2r5ea hif1ab (22868897-) prkchb LOC101884906 six4b]
XP_018969674.2__7962__109100693 LOC109100693 (774) [NC_056616.1: snapc1a LOC109054160 LOC122141104 LOC109100693 (12359870-) LOC109054158 LOC109054156 LOC109054155]
XP_026147030.1__7957__113121035 hif1ab (774) [NC_039262.1: prkchb six4b six1b hif1ab (16017664+) snapc1a LOC113121036 ppp2r5ea]
XP_018970672.2__7962__109101721 LOC109101721 (774) [NC_056591.1: LOC109101722 LOC109101724 LOC109101730 LOC109101721 (11406458-) LOC109101719 LOC109101718 LOC109101717]
XP_026089483.1__7957__113063370 LOC113063370 (781) [NC_039287.1: LOC113063369 LOC113063367 LOC113063368 LOC113063370 (14584005+) LOC113063371 LOC113063372 LOC113062706]
XP_026089492.1__7957__113063374 LOC113063374 (716) [NC_039287.1: LOC113063373 LOC113062706 LOC113063372 LOC113063374 (14667654+) LOC113063376 LOC113062708 LOC113062757]
XP_040556477.1__9031__374177 HIF1A (831) [NC_052536.1: SNAPC1 SYT16 ITPRIP5B HIF1A (53477261-) PRKCH SLC38A6 TRMT5]
NP_001230013.1__9606__3091 HIF1A (850) [NC_000014.9: TMEM30B PRKCH SLC38A6 HIF1A (61695512+) SNAPC1 SYT16 KCNH5]
NP_001300848.1__10090__15251 Hif1a (837) [NC_000078.7: Prkch Tmem30b Slc38a6 Hif1a (73948185+) Snapc1 Syt16 Dbpht2]
1.15

Figure S1. An initial unfiltered phylogenetic tree showing the identification and removal of non-orthologous sequences.

For each leaf branch (except for human and sea urchin branches), we provided the following information: protein sequence accession number, NCBI taxonomy ID, NCBI gene ID, official gene symbol (and **sequence length**), and gene neighbors (starting position and orientation of the target gene were also provided). The light blue background color indicated that the respective clade was selected for further use.

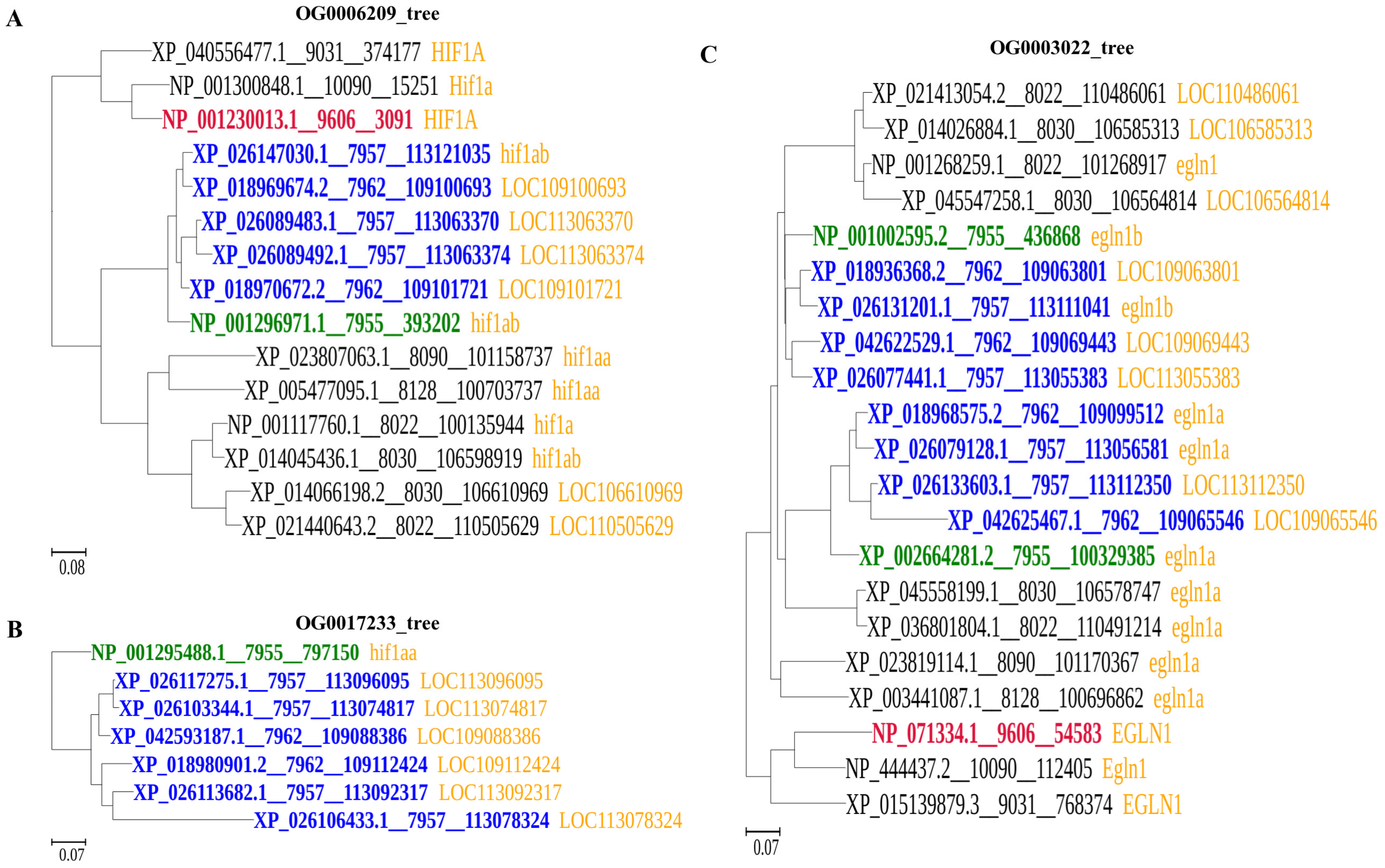
A
OG0006209_tree
XP_040556477.1__9031__374177 HIF1A
NP_001300848.1__10090__15251 Hif1a
NP_001230013.1__9606__3091 HIF1A
XP_026147030.1__7957__113121035 hif1ab
XP_018969674.2__7962__109100693 LOC109100693
XP_026089483.1__7957__113063370 LOC113063370
XP_026089492.1__7957__113063374 LOC113063374
XP_018970672.2__7962__109101721 LOC109101721
NP_001296971.1__7955__393202 hif1ab
XP_023807063.1__8090__101158737 hif1aa
XP_005477095.1__8128__100703737 hif1aa
NP_001117760.1__8022__100135944 hif1a
XP_014045436.1__8030__106598919 hif1ab
XP_014066198.2__8030__106610969 LOC106610969
XP_021440643.2__8022__110505629 LOC110505629
0.08
B
OG0017233_tree
NP_001295488.1__7955__797150 hif1aa
XP_026117275.1__7957__113096095 LOC113096095
XP_026103344.1__7957__113074817 LOC113074817
XP_042593187.1__7962__109088386 LOC109088386
XP_018980901.2__7962__109112424 LOC109112424
XP_026113682.1__7957__113092317 LOC113092317
XP_026106433.1__7957__113078324 LOC113078324
0.07
C
OG0003022_tree
XP_021413054.2__8022__110486061 LOC110486061
XP_014026884.1__8030__106585313 LOC106585313
NP_001268259.1__8022__101268917 egln1
XP_045547258.1__8030__106564814 LOC106564814
NP_001002595.2__7955__436868 egln1b
XP_018936368.2__7962__109063801 LOC109063801
XP_026131201.1__7957__113111041 egln1b
XP_042622529.1__7962__109069443 LOC109069443
XP_026077441.1__7957__113055383 LOC113055383
XP_018968575.2__7962__109099512 egln1a
XP_026079128.1__7957__113056581 egln1a
XP_026133603.1__7957__113112350 LOC113112350
XP_042625467.1__7962__109065546 LOC109065546
XP_002664281.2__7955__100329385 egln1a
XP_045558199.1__8030__106578747 egln1a
XP_036801804.1__8022__110491214 egln1a
XP_023819114.1__8090__101170367 egln1a
XP_003441087.1__8128__100696862 egln1a
NP_071334.1__9606__54583 EGLN1
NP_444437.2__10090__112405 Egln1
XP_015139879.3__9031__768374 EGLN1
0.07

Figure S2. Cross-validation of orthology assignments for HIF-1α and PHD2 using OrthoFinder.

OrthoFinder v2.5.5 was run with the -S diamond_ultra_sens option on the complete proteomes of ten species. Three orthogroups relevant to the HIF pathway were recovered. (**A**) Gene tree of the orthogroup containing euteleost Hif-1α and cyprinid Hif-1αb. (**B**) Gene tree of the orthogroup containing exclusively the divergent cyprinid Hif-1αa paralogs. (**C**) Gene tree of the orthogroup containing Phd2 sequences. Together, panels A and B recapitulate the full set of HIF-1α sequences identified by our manual phylogeny-based approach, while panel C is fully congruent with our manual PHD2 analysis. The separation of cyprinid Hif-1αa into a distinct orthogroup by OrthoFinder illustrates the limited sensitivity of automated tools in resolving highly divergent paralogs, in contrast to our phylogeny-informed synteny-guided strategy.

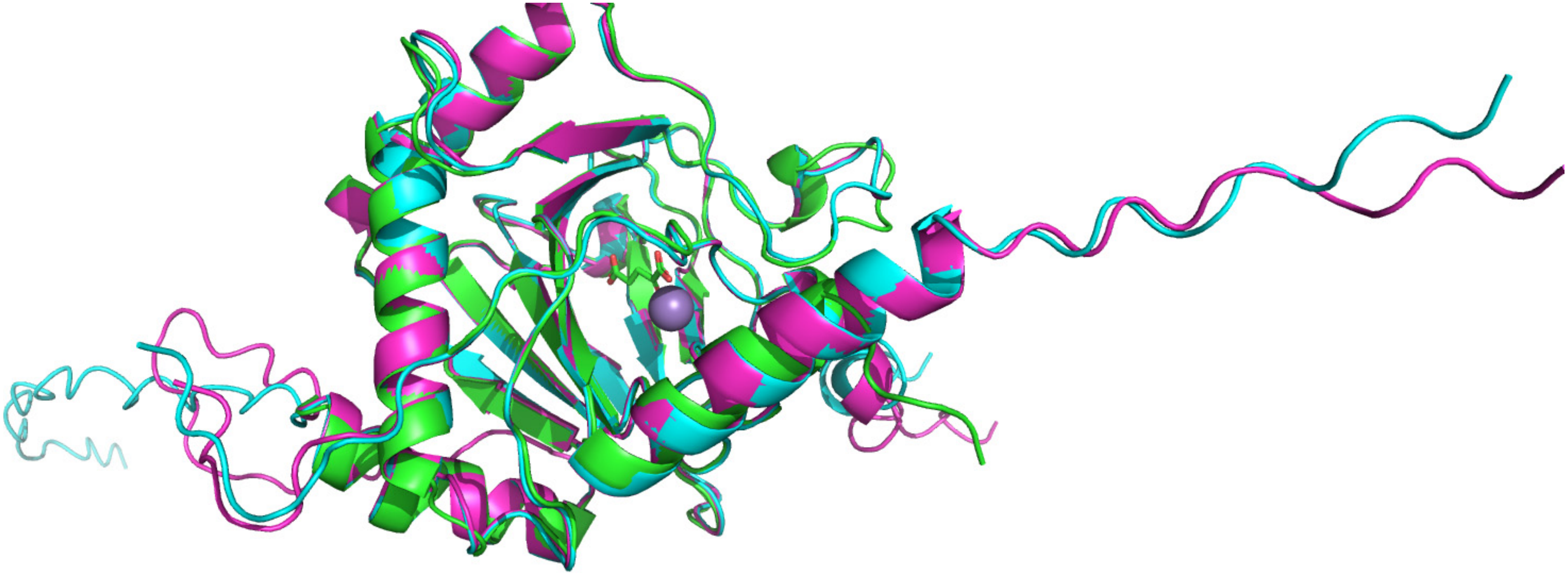

Figure S3. Structural alignment of AlphaFold-predicted Phd2.Hif-1α$_{CODD}$ complexes with the crystallographic template.

The Phd2 domains of the AlphaFold-predicted complexes (rainbow trout Phd2a.Hif-1α$_{CODD}$, **cyan**; Nile tilapia Phd2.Hif-1α$_{CODD}$, **magenta**) were superimposed onto the PHD2 component of the template crystal structure (PDB ID: 5L9B, **green**). The aligned structures show essentially identical folds within the catalytic centers, including the 2-oxoglutarate (2OG) and Mn(II) binding pockets. The root-mean-square deviation (RMSD) for all active-site residues was < 1 Å (actual values < 0.4 Å in the displayed alignment), demonstrating that the predicted binding-site geometries are indistinguishable from the experimental template. CODD, C-terminal oxygen-dependent degradation domain.

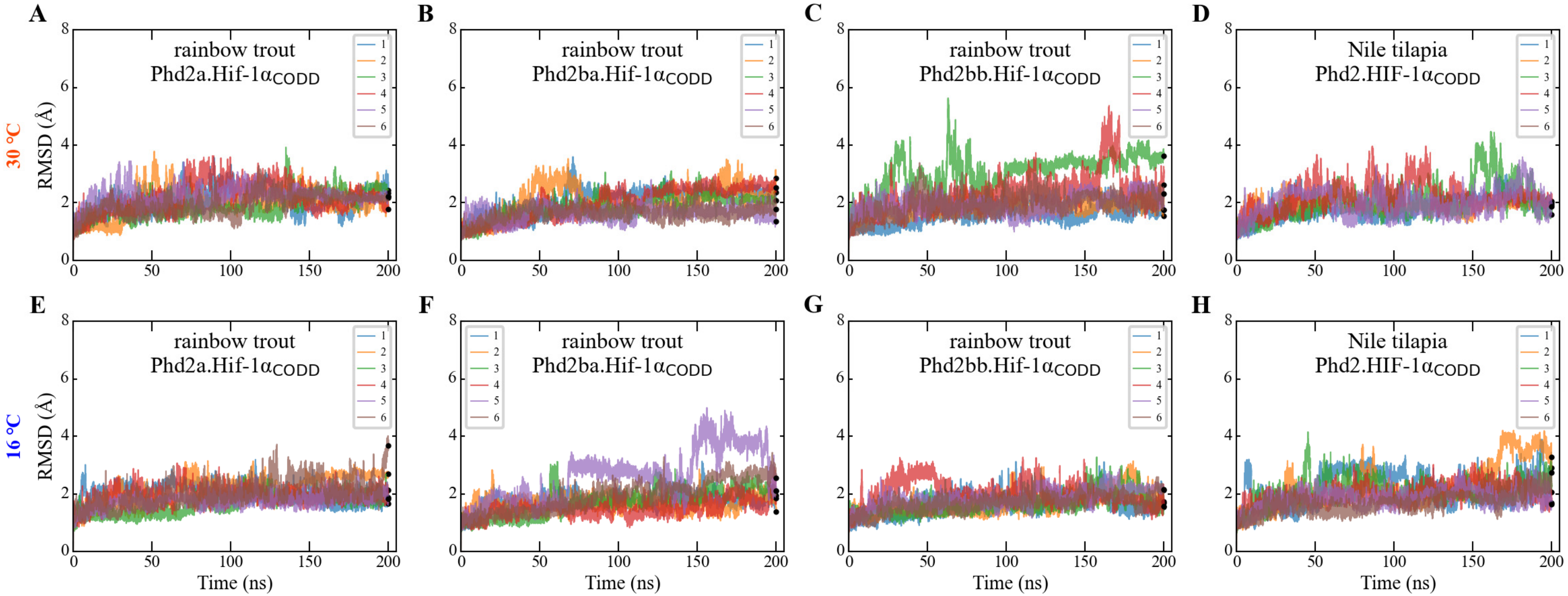

Figure S4. Cα RMSD of Phd2 proteins during MD simulations.

Root mean square deviation (RMSD) of backbone Cα atoms of the Phd2 chain over the 200 ns production trajectories for all simulated complexes. Each panel shows six independent replicas (colored lines 1–6). The panels correspond to the same complexes and conditions as in Figure 3E–L. In most cases, the RMSD values plateau early in the simulations, indicating overall conformational stability of the Phd2 protein throughout the analysis window.

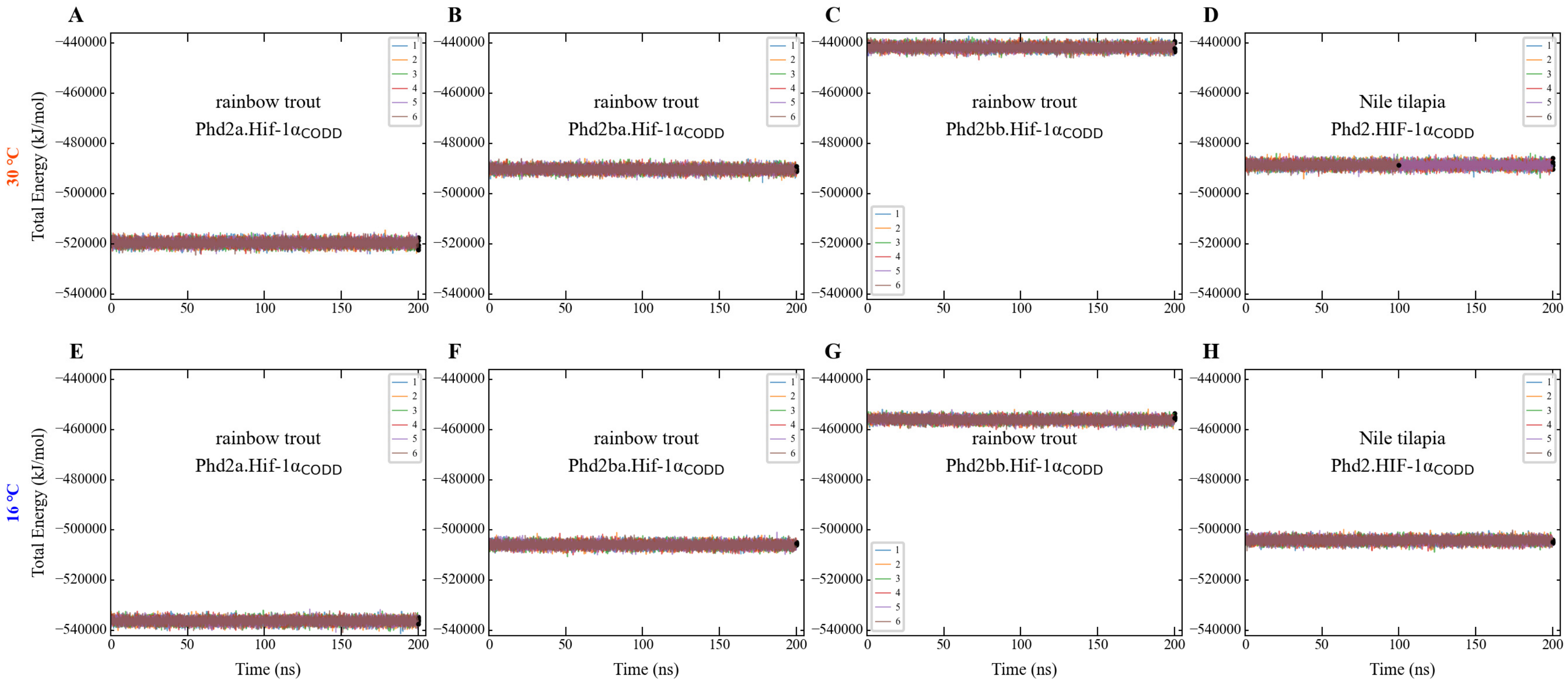

Figure S5. Total energy stabilization during MD simulations.

Total energy (potential + kinetic) as a function of simulation time for all Phd2-Hif-1α CODD complexes. Each panel displays six independent replicas. The energy traces fluctuate stably around a constant mean value for all systems, confirming that thermodynamic equilibrium was achieved and maintained during the 200 ns production runs. Panels are arranged identically to Figure 3E–L.

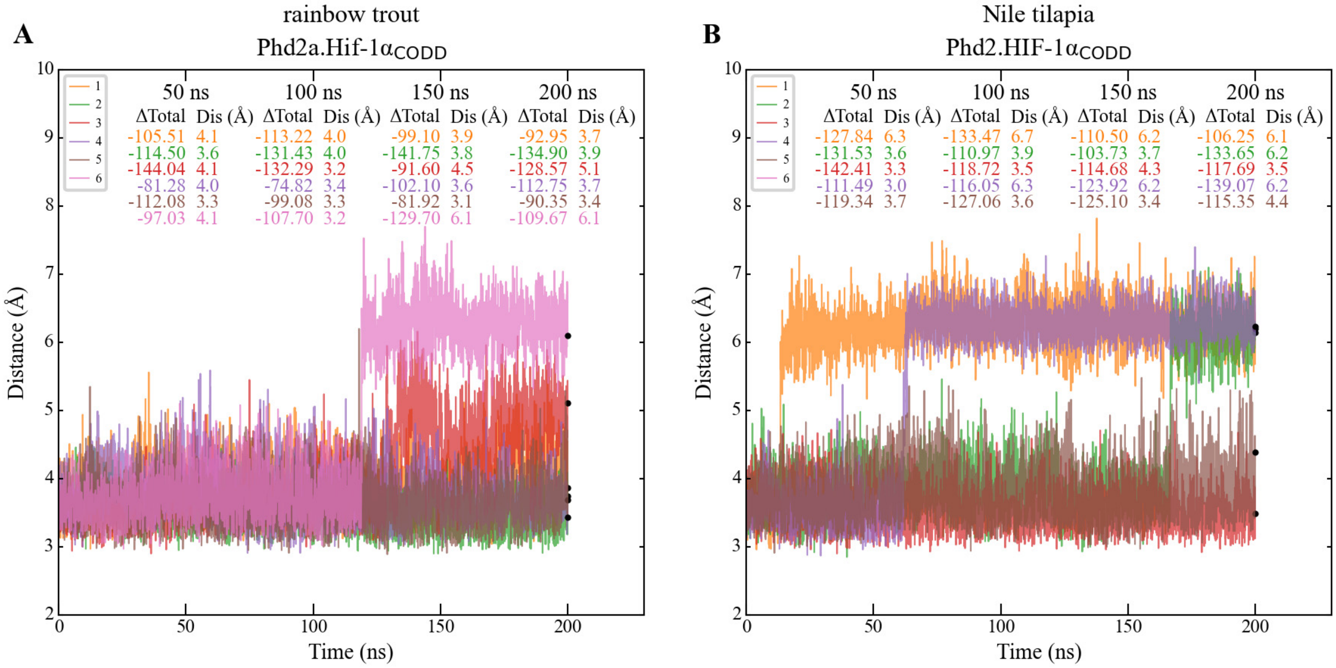

Figure S6. Relationship between calculated binding free energy and CG–O1 distance over time.

Binding free energy was calculated using gmx_MMPBSA with PB solvation models for representative trajectories. Subfigures A and B correspond to the simulations shown in main text Figure 3E and 3H, respectively. The lack of a clear trend underscores the challenge of using thermodynamic endpoints to quantify such highly dynamic interactions. Each ΔTotal value (in kcal/mol) is the average of a 5 ns trajectory.

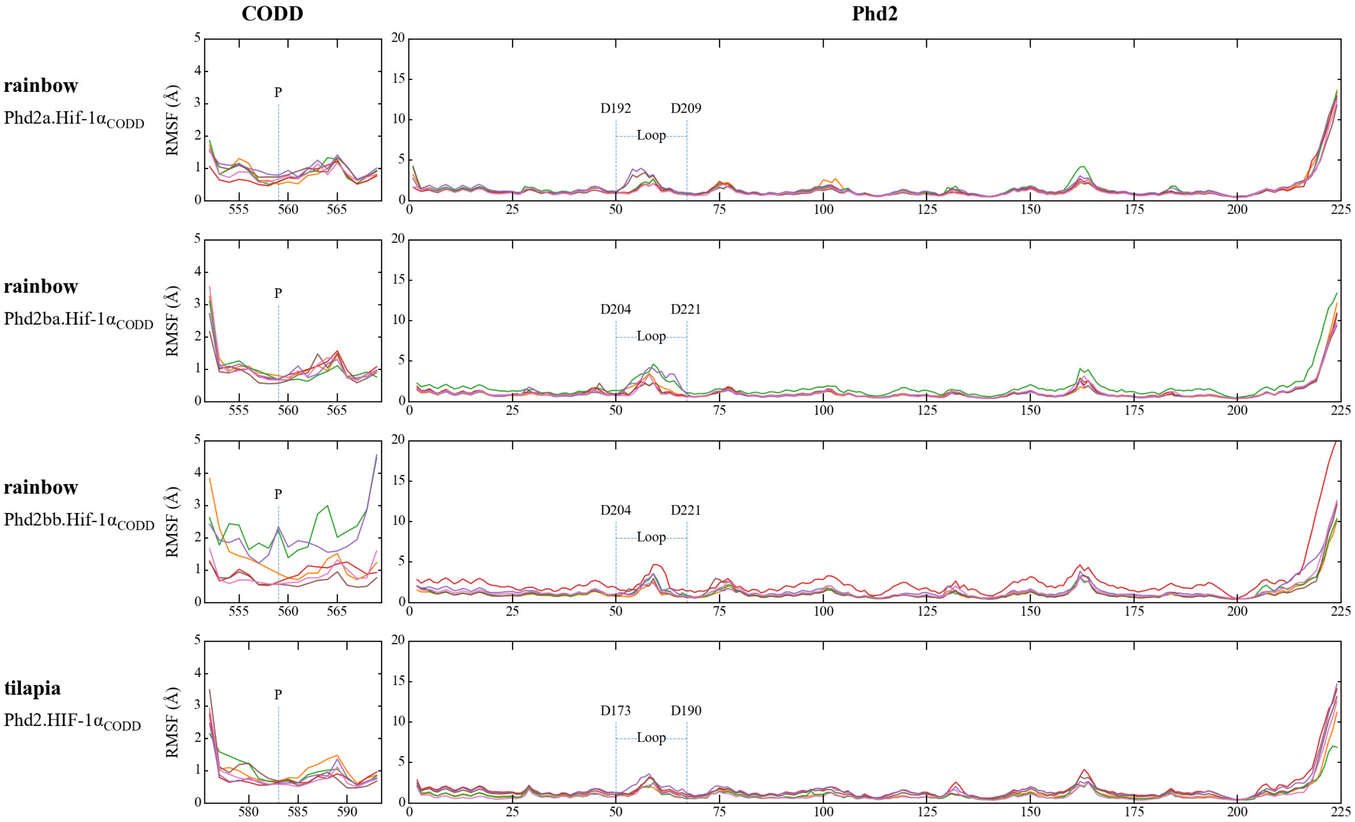

Figure S7. Root mean square fluctuation (RMSF) of Cα atoms in CODD peptides and Phd2.

RMSF profiles are shown for all simulation replicas at 30°C. The Phd2 loop region exhibits high flexibility in all complexes. The X-axis for CODD peptides shows residue positions in their respective sequences; for Phd2, it shows the residue order in selected regions of aligned sequences (exact regions were shown in Table S1).
